# Methods matter: considering locomotory mode and respirometry technique when estimating metabolic rates of fishes

**DOI:** 10.1093/conphys/cow008

**Published:** 2016-03-23

**Authors:** Jodie L. Rummer, Sandra A. Binning, Dominique G. Roche, Jacob L. Johansen

**Affiliations:** 1 Australian Research Council Centre of Excellence for Coral Reef Studies, James Cook University, Townsville, QLD 4811, Australia; 2 Australian Research Council Centre of Excellence for Coral Reef Studies, Division of Evolution, Ecology and Genetics, Research School of Biology, The Australian National University, Canberra, ACT 0200, Australia; 3 Éco-Éthologie, Institut de Biologie, Université de Neuchâtel, Neuchâtel 2000, Switzerland; 4 Marine Science Institute, University of Texas at Austin, Port Aransas, TX 78373, USA

**Keywords:** Body–caudal fin swimming, carangiform, circular swimming chamber, labriform, median–paired fin swimming, oxygen consumption rate

## Abstract

Respirometry is frequently used to estimate metabolic rates and examine organismal responses to environmental change. Although a range of methodologies exists, it remains unclear whether differences in chamber design and exercise (type and duration) produce comparable results within individuals and whether the most appropriate method differs across taxa. We used a repeated-measures design to compare estimates of maximal and standard metabolic rates (MMR and SMR) in four coral reef fish species using the following three methods: (i) prolonged swimming in a traditional swimming respirometer; (ii) short-duration exhaustive chase with air exposure followed by resting respirometry; and (iii) short-duration exhaustive swimming in a circular chamber. We chose species that are steady/prolonged swimmers, using either a body–caudal fin or a median–paired fin swimming mode during routine swimming. Individual MMR estimates differed significantly depending on the method used. Swimming respirometry consistently provided the best (i.e. highest) estimate of MMR in all four species irrespective of swimming mode. Both short-duration protocols (exhaustive chase and swimming in a circular chamber) produced similar MMR estimates, which were up to 38% lower than those obtained during prolonged swimming. Furthermore, underestimates were not consistent across swimming modes or species, indicating that a general correction factor cannot be used. However, SMR estimates (upon recovery from both of the exhausting swimming methods) were consistent across both short-duration methods. Given the increasing use of metabolic data to assess organismal responses to environmental stressors, we recommend carefully considering respirometry protocols before experimentation. Specifically, results should not readily be compared across methods; discrepancies could result in misinterpretation of MMR and aerobic scope.

## Abstract (precis)

We estimated maximum metabolic rates (MMR) in four steady-swimming coral reef fishes - two body-caudal and two median-paired fin swimmers - using three different techniques. Differences between MMR estimates were not due to locomotory mode, but rather the method used to induce maximum performance, with swimming respirometry resulting in the highest estimates.

## Introduction

The growing fields of ecological and conservation physiology (Wikelski and Cooke, 2006; Cooke *et al*., 2013) aim to understand the mechanisms underpinning the behaviour and fitness of organisms in changing environments. As human impacts on global ecosystems continue to increase, greater emphasis will be placed on research that aims to understand whether and how organisms respond and adapt to anthropogenic and environmental stressors (Pörtner and Farrell, 2008; Alcaraz *et al*., 2013). Choosing appropriate methodologies to study a given species or system is critical to ensure that data are robust and comparisons among studies valid.

The performance of an organism over a range of activities can be tightly linked to oxygen utilization. Respirometry is a tool commonly used to measure oxygen consumption rates ($\dot{M}O_{2}$) and estimate the metabolic performance of an organism at rest, during exposure to stressors or while performing different locomotory activities. Two important physiological parameters describe the upper and lower bounds of an organism's capacity to metabolize energy. Maximal metabolic rate (MMR) is the maximal amount of energy that can be metabolized aerobically by an organism and can be estimated by measuring an organism's $\dot{M}O_{2}$ during or immediately after exhaustive exercise ($\dot{M}O_{2\mathrm{Max}}$; Norin and Clark, 2016). In contrast, standard metabolic rate (SMR) is the minimal amount of energy required for maintenance and is estimated by measuring $\dot{M}O_{2}$ in a post-absorptive, resting state ($\dot{M}O_{2\mathrm{Min}}$; Nelson and Chabot, 2011; Chabot *et al.*, 2016). The difference between $\dot{M}O_{2\mathrm{Max}}$ and $\dot{M}O_{2\mathrm{Min}}$ is the absolute or total scope for aerobic activity (aerobic scope; AS), which is, in essence, the capacity for aerobic metabolism, in excess of basic maintenance costs, for activities essential to support biological fitness, such as swimming, feeding and reproduction (Brett, 1964). Respirometry can therefore provide essential information about the metabolic performance of an organism and is thus rapidly becoming more widely used (Clark *et al.,* 2013; Norin and Clark, 2016).

A particularly interesting model system is fish, where experimental techniques in respiratory physiology have been widely used for nearly a century and increasingly over recent decades (Webb, 1975; Steffensen *et al.*, 1984, 1989; Svendsen *et al*., 2016). Many of these techniques are used extensively today in ecological and conservation physiology studies (Nilsson *et al*., 2007, 2009; Donelson *et al*., 2011; Couturier *et al*., 2013; McLeod *et al*., 2013; Rummer *et al*., 2013, 2014 Trappett *et al*., 2013; Binning *et al.*, 2014).

**Figure 1:**
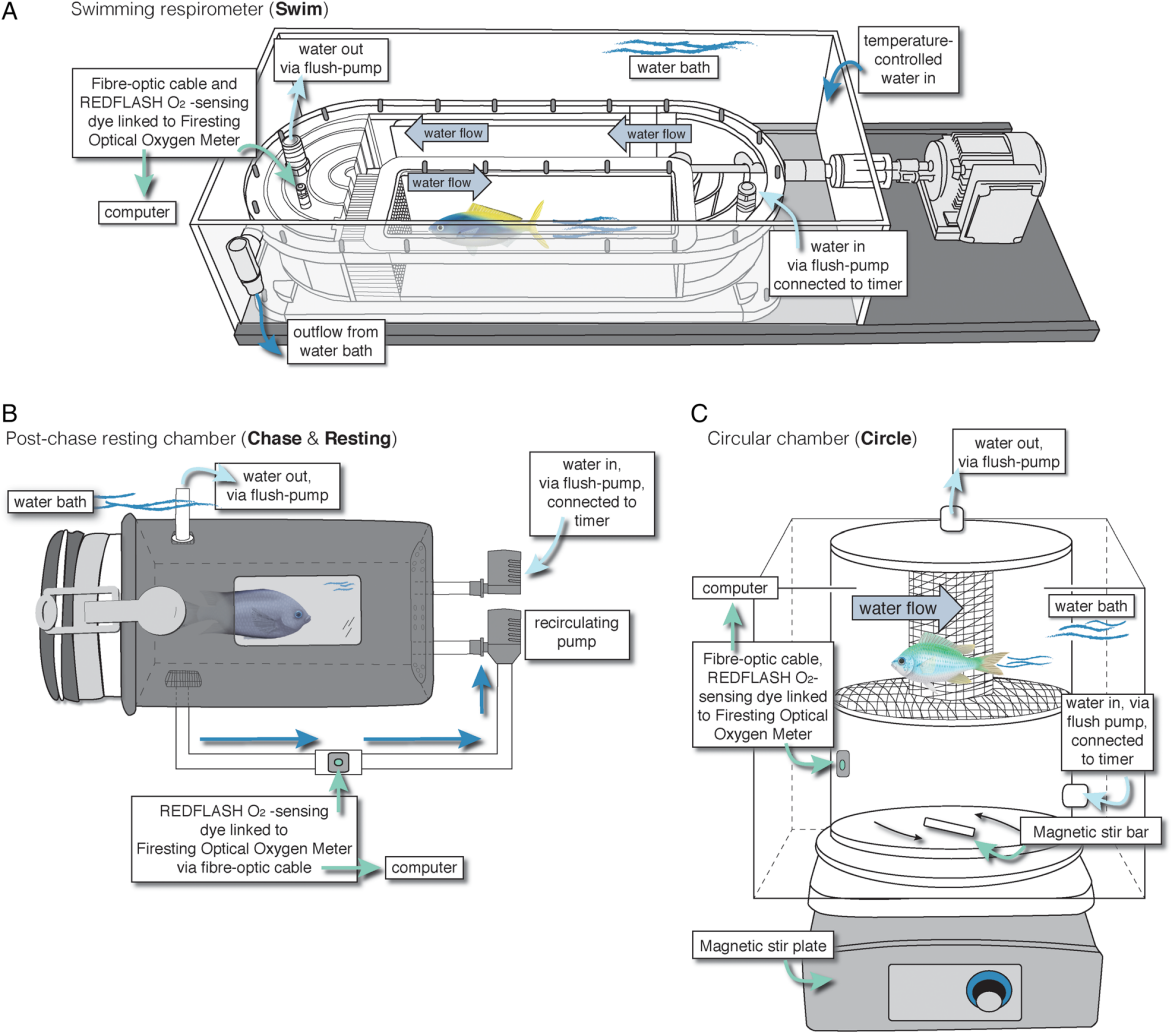
Schematic diagrams of the three respirometers used in this study: a swimming respirometer (**A**), a resting respirometer (post exhaustive chase; **B**) and a circular chamber respirometer (**C**).

The most established method to estimate MMR in fish uses a treadmill-like swimming respirometry chamber (hereinafter, swimming respirometry), where individuals swim against near-laminar water flow at incrementally increased speeds until fatigue is reached (Fig. 1A; Brett, 1964). Using this protocol, SMR can be estimated indirectly by extrapolating the non-linear $\dot{M}O_{2}$–swimming speed relationship to zero swimming speed (Steffensen *et al*., 1984; Steffensen, 2005; Roche *et al*., 2013).

A second common method uses an exhaustive chase protocol (commonly referred to as the ‘stick method’), during which fish are first chased to exhaustion (some protocols also incorporate air exposure) and then placed into a resting respirometer that closely matches the fish's size, such that active swimming is restricted (Fig. 1B; Ferguson and Tufts, 1992; Donaldson *et al*., 2010; Clark *et al*., 2012, 2013; Roche *et al*., 2013). The $\dot{M}O_{2\mathrm{Max}}$ calculated immediately after this exhaustive exercise (which includes repayment of the oxygen debt resulting from anaerobic activity) is used as an indirect estimate of MMR. After recovery from the chase, SMR can be estimated directly once the fish has fully recovered from exercise (6–24 h depending on species; Milligan and Wood, 1986; Milligan, 1996; Milligan *et al.*, 2000; Chabot *et al.*, 2016).

A third and more recent method uses a circular chamber and a magnetic stir-bar to create a vortex-like water flow, against which a fish must swim facing either clockwise or anticlockwise (Munday *et al.*, 2009; Gardiner *et al.*, 2010; Couturier *et al*., 2013; Rummer *et al*., 2013; Trappett *et al.*, 2013; Pope *et al.*, 2014; hereinafter, circular chamber; Fig. 1C, online supplementary material, Fig. S1, but referred to as a ‘swim respirometer’ by Nilsson *et al*., 2007). The circular chamber combines aspects of both swimming and resting respirometers, but instead the fish swims in tight circles. As in a traditional swimming respirometer, this method allows direct estimates of MMR during exertion. In contrast to traditional swimming respirometry, direct estimates of SMR can also be obtained, as in resting chambers, when the revolutions of the stir-bar are reduced to a level that gently mixes the water but does not induce swimming. All three methods have been used interchangeably to estimate metabolic performance in fishes. However, studies suggest that the choice of method could significantly affect the accuracy (Reidy *et al*., 1995; Roche *et al*., 2013; Norin and Clark, 2016; Svendsen *et al*., 2016) and, perhaps, repeatability of MMR and SMR estimates.

**Figure 2:**
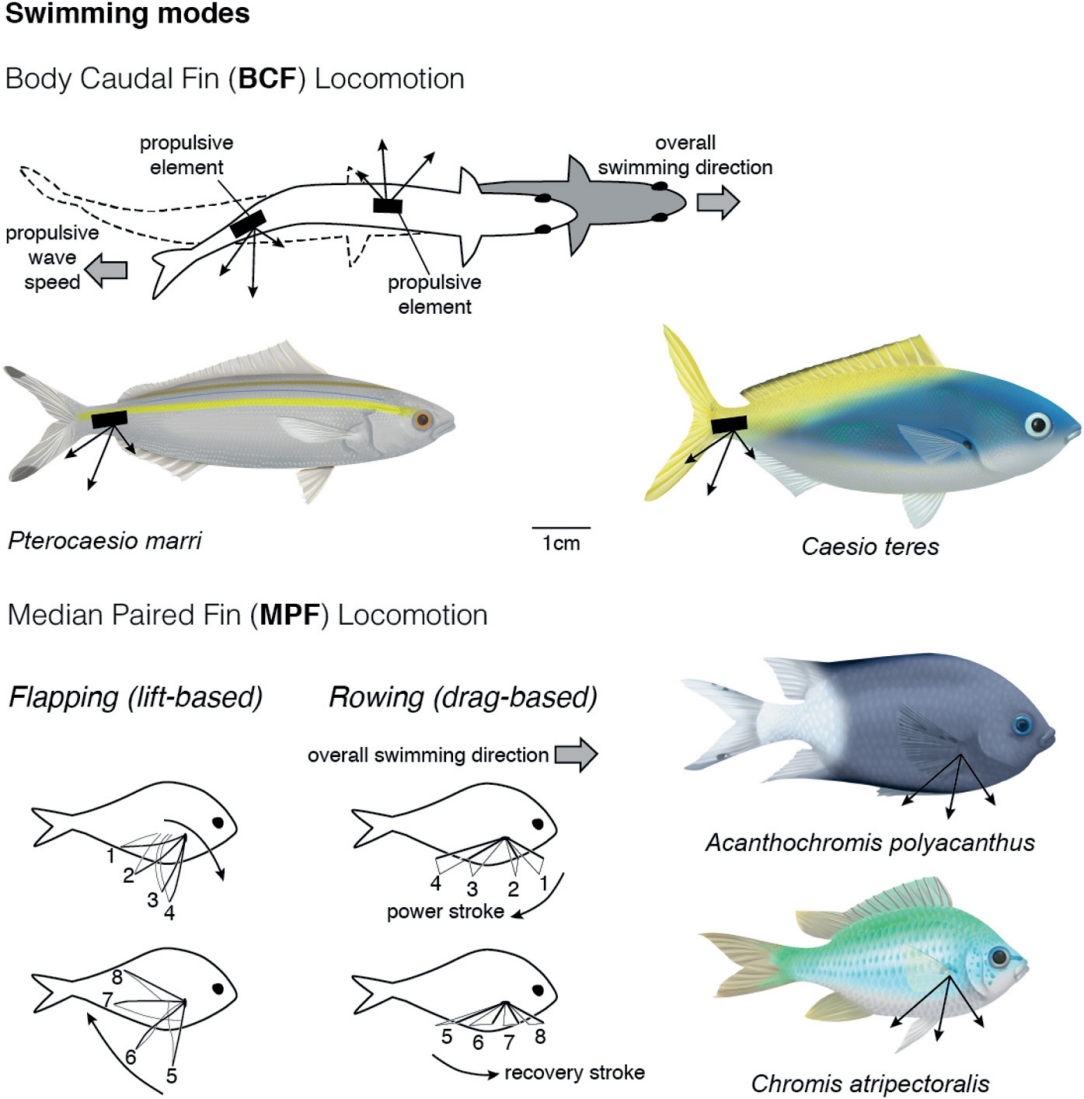
Schematic diagram of thrust generation and propulsion produced by body–caudal fin (BCF) and median–paired fin (MPF) swimming. This study focused specifically on BCF and lift-based MPF species.

Swimming performance is crucial for nearly every aspect of a fish's ecology, including predator–prey interactions, reproductive behaviour and habitat selection (Breder, 1926; Webb, 1975, 1984; Blake, 2004), and engages a variety of body regions and fin appendages (referred to hereinafter as swimming modes; Breder, 1926). Functional differences in swimming modes may have important implications for determining the best method of obtaining accurate metabolic rate estimates. For instance, body–caudal fin (BCF) swimming (e.g. carangiform) is powered by movements of the caudal (tail) fin and the posterior half of the body (Fig. 2) and is often (but not always) coupled with a long, streamlined body ideal for long-distance and fast-propulsive swimming in open environments (Videler, 1993; Blake, 2004). Alternatively, median–paired fin (MPF; e.g. labriform; pectoral fin) swimming is powered by movements of the median or paired fins, such as the pectoral or dorsal–ventral fins, while maintaining a rigid body (Fig. 2), and is thought to promote manoeuvrability (Korsmeyer *et al*., 2002). These two swimming modes differ kinematically in how thrust is produced and physiologically in terms of which muscles are used during swimming (Blake, 2004). Although BCF swimming is used by many species during high-speed escapes or chases, species from many families of coral reef fishes (e.g. Acanthuridae, Labridae, Pomacentridae and Scaridae) regularly use an MPF swimming mode during daily activities. Currently, no study has evaluated whether functional differences in routine swimming mode affect which respirometry method should be used to obtain the best metabolic rate estimates.

Previous studies that compare methods or calculations for estimating metabolic rates in fishes are restricted to one or two species and/or a single swimming mode (e.g. Reidy *et al*., 1995; Schurmann and Steffensen, 1997; Korsmeyer *et al*., 2002; Roche *et al*., 2013). This limits our ability to generalize results across taxa with different swimming modes, swimming durations and/or life histories. Additionally, time-sensitive and field-based studies would benefit from more portable and faster protocols for estimating MMR and SMR. The chase and circular chamber methods may therefore provide more rapid options for estimating MMR than traditional swimming respirometry, which is expensive and can require upwards of 12 h per individual. Therefore, the aims of this study were as follows: (i) to compare metabolic rate estimates obtained using three common methods, i.e. a prolonged swim trial using a swimming respirometer, a short-duration exhaustive chase protocol with air exposure followed by resting respirometry, and a short-duration exhaustive swimming trial in a circular chamber followed by resting respirometry; and (ii) to determine whether metabolic rate estimates obtained with each method vary among four fish species, all of which are prolonged swimmers but exhibit different swimming modes (BCF and MPF).

## Materials and methods

### Experimental animals

We used four species of coral reef fishes, all of which are abundant on the Great Barrier Reef, Australia, to explore differences in MMR and SMR estimates across three respirometry methods and two swimming modes. We chose two predominantly BCF swimming caesionid (fusilier) species [*Pterocaesio marri* (sample size *n* = 5; standard length, SL, 85.2 ± 11.4 mm; wet mass 9.89 ± 1.86 g; means ± SD) and *Caesio teres* (*n* = 11; 82.2 ± 6.7 mm; 15.07 ± 4.46 g)] that form mixed-species shoals that cruise mid-water along the reef edge (Fig. 2). We also chose two predominantly MPF swimming pomacentrid (damselfish) species [*Acanthochromis polyacanthus* (*n* = 11; 74.2 ± 4.8 mm; 16.88 ± 3.38 g) and *Chromis atripectoralis* (*n* = 10; 62.5 ± 2.2 mm; 8.10 ± 1.51 g)] that are site attached as adults and territorial (Randall *et al*., 1997; Fig. 2). The MPF swimmers we chose generate lift-based thrust by flapping their fins, in contrast to other MPF swimmers that produce drag-based thrust by rowing their fins like paddles, a behaviour better suited for low-speed manoeuvring (Vogel, 1994; Walker and Westneat, 2002; Binning and Roche, 2015; Fig. 2). All four species co-occur on the mid-shelf reef crest and feed primarily on plankton in the water column (Randall *et al*., 1997). Despite differences in their habitat use, all four species are considered steady/prolonged swimmers.

Fishes were collected from reef crest sites around Lizard Island (14°40′08″S; 145°27′34″E) using monofilament barrier and hand nets under Marine Parks Permit #G10/33239.1. Fishes were maintained in flow-through aquaria directly from the reef at the research station laboratory at ambient temperature (∼27°C) and fed to satiation daily (NRD pellets; INVE Aquaculture, Salt Lake City, UT, USA) until being transferred to the James Cook University Marine Aquarium Research Facilities Unit in Townsville, Queensland, Australia, ∼14 days after collection. At James Cook University, fishes were evenly distributed among five aquaria supplied with well-aerated seawater (28.5 ± 0.5°C) and fed to satiation daily (NRD pellets) for a minimum of 19 days before the experiments commenced. Throughout the duration of the project, fishes were maintained under James Cook University Animal Ethics Committee regulations (permit #A1722, approved for this study) according to the Australian Code of Practice for the Care and Use of Animals for Scientific Purposes and the Queensland Animal Care and Protection Act 2001.

Prior to experimental procedures, individual fish were fasted for 36 h in separate aquaria to ensure a post-absorptive state (Niimi and Beamish, 1974). Fishes were tested in the three different respirometry methods in a random order following a repeated-measures design. All experiments were performed between 22 October and 14 November 2012.

### Swimming respirometry

Oxygen consumption rates ($\dot{M}O_{2}$) were measured for solitary individuals swimming in a 4.8 l custom-built Steffensen-type Plexiglas swimming respirometer (Fig. 1A; Steffensen *et al.*, 1984; Johansen and Jones, 2011; Roche *et al.*, 2013), a system that allows oxygen consumption rates and swimming performance to be measured simultaneously while fish are being exercised.

The working section of the swimming chamber was 7.0 cm × 36.0 cm × 7.0 cm (width × length × depth), but the entire respirometer was immersed in a temperature-controlled bath and maintained at 28.5 ± 0.1°C (mean +/− SD) (Fig. 1A). Flow straighteners were used to create laminar flow within the working section, and flow was calibrated from 0 to 86.6 ± 0.5 cm s^−1^ (mean ± SD) using a digital vane wheel flow sensor (Höntzsch GmbH, Waiblingen, Germany; model #ZS30GFE-md20T). Solid blocking effects of the fish in the working section were corrected following Bell and Terhune (1970) and did not exceed 5% for any individual.

At the beginning of each trial, the respirometer was filled with temperature-controlled, filtered, ultraviolet-sterilized, well-aerated seawater. Next, a fish was placed in the working section of the chamber and left to habituate for 4–8 h at a swimming speed of 0.5 BL s^−1^ (body length, taken as the standard length of the fish, per second) until the fish had settled into a continuous swimming rhythm and $\dot{M}O_{2}$ stabilized. Preliminary trials conducted on all four species in the same chamber demonstrated that all reached gait transitions and critical swimming speeds (*U*_crit_) well above 4 BL s^−1^. Specifically, gait transitions occur when MPF swimmers transition from using paired fins to caudal-assisted swimming (*U*_p-c_; Johansen and Jones, 2011; Binning *et al*., 2014) or when BCF swimmers transition from steady swimming to anaerobic burst and coast motions (*U*_burst_; Svendsen *et al*., 2010) as speeds increase. As is common in other fast, steady-swimming coral reef fish species (Johansen and Jones, 2011; Roche *et al.,* 2013), increases in $\dot{M}O_{2}$ are only marginal at swimming speeds <40% of *U*_crit_. Therefore, after the initial habituation period at 0.5 BL s^−1^, swimming protocols commenced at 4 BL s^−1^, which was substantially below *U*_pc_, *U*_burst_, and *U*_crit_ for these species. All fish were swum during daylight hours.

After the 0.5 BL s^−1^ habituation period, the swimming trial commenced by increasing the water flow speed to 4 BL s^−1^ over a period of 4 min. Then, starting at 4 BL s^−1^, $\dot{M}O_{2}$ was measured, and the swimming speed was incrementally increased (increments of 0.5 BL s^−1^) following a standard *U*_crit_ protocol (Jain *et al.*, 1997). Fish swam at each speed for three 8 min cycles (i.e. 24 min at each speed). Each 8 min cycle consisted of a 5 min measurement period and a 3 min flush period to replenish the chamber with filtered, well-aerated seawater. Fish were continuously monitored throughout the swimming trial, and the trial was considered finished when the fish could no longer swim against the flow, being swept downstream onto a retaining grid for >5 s (*U*_crit_; Roche *et al.*, 2013). At this point, the total swimming time and flow speed were recorded, the experiment was terminated, and the fish was returned to its holding tank. Maximal metabolic rate (MMR_Swim_) was estimated from the $\dot{M}O_{2}$ at the maximal swimming speed where fish completed at least one full 8 min cycle.

Critical swimming speed (*U*_crit_) was calculated following Brett (1964): *U*_crit_ = *U* + *U_i_* × (*t*/*t_i_*), where *U* is the penultimate swimming speed before the fish fatigued and stopped swimming (*U*_crit_); *U_i_* is the swimming speed at which the fish was unable to continue swimming (i.e. swimming speed at increment *i*); *t* is the length of time the fish swam at the final swimming speed where fatigue occurred; and *t_i_* is the amount of time fish were swum at each speed interval in the trial (24 min).

### Exhaustive chase protocol and resting respirometry

Individual fish were placed into a 0.6 m (diameter) round aquarium containing well-aerated and temperature-controlled seawater (0.15 m deep) maintained at the same temperature as the respirometer (28.5 ± 0.1°C). Fish were then chased continuously by hand for 3 min, during which time the experimenter would touch the tail of the fish if it slowed down or stopped swimming. All species swam primarily with their caudal fin when chased, repeatedly bursting away from the stimulus. Fish were deemed exhausted when they became unresponsive to chasing, which always occurred by the end of the 3 min chase period. Fish were then scooped into a rubber mesh net and maintained out of the water for 1 min (Clark *et al.*, 2013; Roche *et al*., 2013). After air exposure, fish were immediately placed in a 1615 ml closed-loop, recirculating, resting respirometry chamber submerged in a temperature-controlled water bath (Fig. 1B; reviewed by Clark *et al*., 2013). The $\dot{M}O_{2}$ measurement period commenced within 10 s of placing the fish in the chamber and continued for a period of 5 min. The $\dot{M}O_{2\mathrm{Max}}$ was calculated from the steepest 1 min slope during this 5 min interval and used to estimate MMR (MMR_Chase_). Standard metabolic rate (SMR_Rest_) was estimated from $\dot{M}O_{2}$ values obtained after leaving the fish in the chamber for an additional 6–12 h. This time period was deemed sufficient to ensure that $\dot{M}O_{2}$ stabilized and no longer decreased in each species investigated here (online supplementary material, Fig. S2; also see Roche *et al*., 2013; Rummer *et al*., 2013).

### Circular chamber respirometry

Individual fish were placed into a 2654 ml (17.2 cm internal height, 14.8 cm internal diameter) o-ring sealed cylinder (Perspex, 0.3 cm thickness) connected via tubing to a submersible pump in a temperature-controlled water bath (28.5 ± 0.1°C; Fig. 1C, online supplementary material, Fig. S1). A magnetic stir plate below the chamber activated a stir bar (1 cm × 6 cm) to create a circular water motion in the chamber. The speed of the rotating stir bar was increased over a period of ∼1 min to the maximal speed at which the fish could only just maintain its position in the chamber (Fig. 1C, online supplementary material, Fig. S1; see also Nilsson *et al*., 2007). If the fish could no longer hold position in the chamber, the speed was decreased slightly until it was able to maintain position while swimming. The $\dot{M}O_{2\mathrm{Max}}$ was calculated from the steepest 1 min slope of the change in O_2_ depletion recorded during the first 5–7 min of measuring (between 5 and 10 min used in previous studies; Nilsson *et al*., 2009; Gardiner *et al*., 2010; Couturier *et al*., 2013; Rummer *et al*., 2013), which corresponds to MMR_Circle_. The chamber was then flushed, and the rotational speed of the stir bar was decreased to a minimal speed, at which the fish was able to stop swimming and rest on the false mesh bottom (see Fig. 1C). Similar to the chase protocol, SMR was estimated from $\dot{M}O_{2}$ values obtained after leaving the fish in the circular chamber for an additional 6–12 h until $\dot{M}O_{2}$ stabilized and no longer decreased (SMR_Circle_; online supplementary material, Fig. S2).

### General respirometry information and calculations

We used intermittent-flow respirometry (Steffensen, 1989; Svendsen *et al.*, 2016) for all three methods. A digital relay timer (MFRT-1 Multi Function Recycling Timer; Xiamen SUPERPRO Technology Co., Ltd, Xiamen, Fujian, China) was connected to submersible pumps to repeat an 8 min cycle that began with a 5 min measurement period followed by a 3 min flush period. The measurement period was short enough to ensure that oxygen within the chambers remained above 80% air saturation at all times. This is important to ensure that oxygen consumption rates are not influenced by the adrenergic stress response or other metabolic changes associated with hypoxia (Hughes, 1973; Tetens and Lykkeboe, 1985; Boutilier *et al*., 1988). The flush period was long enough to ensure that oxygen levels returned to 100% air saturation. During the experiments, test fish were shielded from outside stimuli by a dark cloth to avoid unwanted stress. However, there was a small viewing window in each chamber for the researcher to check on the fish and so that the fish could still experience photoperiod (Fig. 1B). Temperature- and barometric pressure-compensated O_2_ concentration (in milligrams per litre) in the water were continuously measured at 0.5 Hz using oxygen-sensitive REDFLASH dye on contactless spots (2 mm) adhered to the inside of each chamber. Spots were linked to a Firesting Optical Oxygen Meter (Pyro Science e. K., Aachen, Germany) via fibre-optic cables. Each sensor was calibrated using fully aerated seawater (as 100%) prior to each trial and with sodium sulphite (as zero) weekly or as needed.

Text files were imported into LabChart v. 6.1.3 (ADInstruments, Dunedin, New Zealand), and $\dot{M}O_{2}$ (in milligrams of O_2_ per kilogram per hour) was calculated as the slope of the linear regression of oxygen concentration decline over time during the measurement period of each cycle using the following equation:

$$\dot{M}O_{2}=SV_{\mathrm{resp}}M^{-1},$$

modified from (Bushnell *et al*., 1994; Schurmann and Steffensen, 1997), where *S* is the slope (in milligrams of O_2_ per litre per secondl), *V*_resp_ is the volume of the respirometer minus the volume of the fish (in litres), and *M* is the mass of the fish (in kilograms). We subtracted the proportional background O_2_ consumption rate (measured as O_2_ depletion in the empty respirometer before and after each trial, assumed linear) from each $\dot{M}O_{2}$ measurement. To limit background respiration rates to <5% of a fish's SMR, chambers and pumps were rinsed daily with a 10% bleach solution and fresh water and allowed to dry overnight prior to commencing trials on the next day. The SMR (in milligrams of O_2_ per kilogram per hour) was estimated from the average of the lowest 10% of $\dot{M}O_{2}$ values (Clark *et al*., 2013; Rummer *et al*., 2013, 2014).

### Statistical analyses

We used linear mixed-effects models (LMM; ‘nlme’ package in R) to compare estimates of MMR and SMR obtained with different respirometry methods on the same individuals. This repeated-measures design minimized inter-individual variation in metabolic rate estimates. Species and respirometry method were specified as fixed factors, and fish identity was included as a random factor to control for the non-independence of data points collected using the same individual (Bolker *et al*., 2009). *Post hoc* multiple comparisons were done using the R function ‘ghlt’ in the package ‘multcomp’. This model also allowed for individuals to be included even if they did not complete all three methods. We used two general linear models (LM) and Tukey's tests to examine differences in absolute and relative *U*_crit_ among swimming modes and species. Diagnostic plots were used to ensure that the data met the assumptions of the models. Non-significant interactions were removed for model simplification and fit. Analyses were performed in R v3.0.2 (R Core Team, 2013).

## Results

**Figure 3:**
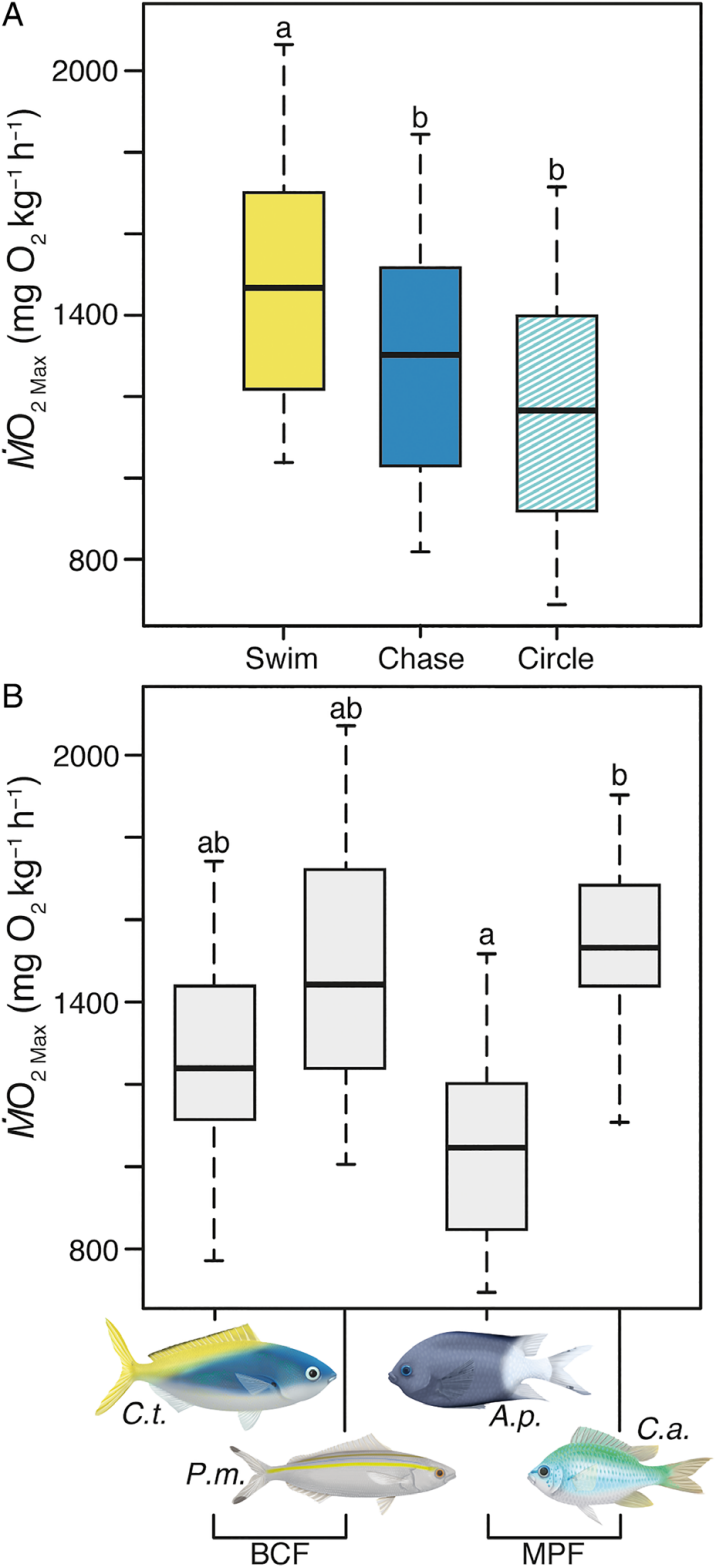
Boxplots showing median and inter-quartile range of (**A**) maximal metabolic rates (MMR; estimated from the highest value of oxygen consumption rate, $\dot{M}O_{2\mathrm{Max}}$) using three respirometry methods, a critical swimming speed trial in a traditional swimming respirometer (Swim), an exhaustive chase protocol followed by 1 min air exposure (Chase), and an exhaustive swim trial in a circular chamber with a stir bar (Circle), for all species combined that completed at least one method and (**B**) MMR for all fishes, by species, that completed at least one method. *Caesio teres* (*C.t.*) and *Pterocaesio marri* (*P.m.*) are body–caudal fin (BCF) swimmers. *Acanthochromis polyacanthus* (*A.p*) and *Chromis atripectoralis* (*C.a.*) are median–paired fin (MPF) swimmers. Same letters indicate no significant differences (α = 0.05).

Estimates of MMR differed significantly between respirometry methods (LMM, *F*_2,62_ = 11.38, *P *< 0.001; Fig. 3A and Table 1) and fish species (LMM, *F*_3,33_ = 4.13, *P *= 0.014; Fig. 3B and Table 1). However, the effect of method was the same across all species (species* *× method interaction not significant, *F*_6,56_ = 0.74, *P *= 0.62; online supplementary material, Fig. S3A). The value of MMR_Swim_ was consistently higher than MMR_Chase_ (*z* = 2.95, *P* < 0.01) and MMR_Circle_ (*z* = 4.79, *P* < 0.001) for all species (BCF and MPF swimmers; Fig. 3A). Specifically, MMR_Swim_ was 20% higher than MMR_Chase_ based on model predictions computed with the R package effects (Fox *et al*., 2014); this difference ranged from 6.3 to 35.3% across the four species. Likewise, MMR_Swim_ was 25% higher than MMR_Circle_; this difference ranged from 15.5 to 38.3% across the four species. On average, MMR_Chase_ was numerically higher than MMR_Circle_, but this difference was not statistically significant (*z* = 1.84, *P* = 0.15; Fig. 3A).

**Table 1:** Estimates of maximal metabolic rate and standard metabolic rate for all fishes that completed at least one of the three different respirometry methods

Species		MMR (mg O_2_ kg^−1^ h^−1^)			SMR (mg O_2_ kg^−1^ h^−1^)	
		Swim	Chase	Circle	Rest	Circle
*Pterocaesio marri*	Mean	1794.3	1376.4	1308.1	223.3	257.9
(*n* = 5)	SEM	336.6	182.5	254.8	40.3	24.6
*Caesio teres*	Mean	1299.4	1277.9	1175.8	167.7	176.5
(*n* = 11)	SEM	59.4	169.8	102.5	31.3	17.5
*Acanthochromis polyacanthus*	Mean	1150.7	1048.1	974.4	143.0	145.1
(*n* = 11)	SEM	112.3	164.2	80.4	26.1	7.2
*Chromis atripectoralis*	Mean	1768.0	1556.7	1312.8	221.0	154.5
(*n* = 10)	SEM	112.2	134.7	104.5	35.9	10.2

Methods included a critical swimming speed trial in a Steffensen-type swimming respirometer (Swim), an exhaustive chase protocol followed by 1 min air exposure (Chase), and an exhaustive swim trial in a circular chamber with a stir bar (Circle). MMR, maximal metabolic rate; and SMR, standard metabolic rate. Values are group means; see the Results section and online supplementary material, Fig. S1 for model predictions that account for repeated measures on the same individuals (i.e. blocking by individual).

**Figure 4:**
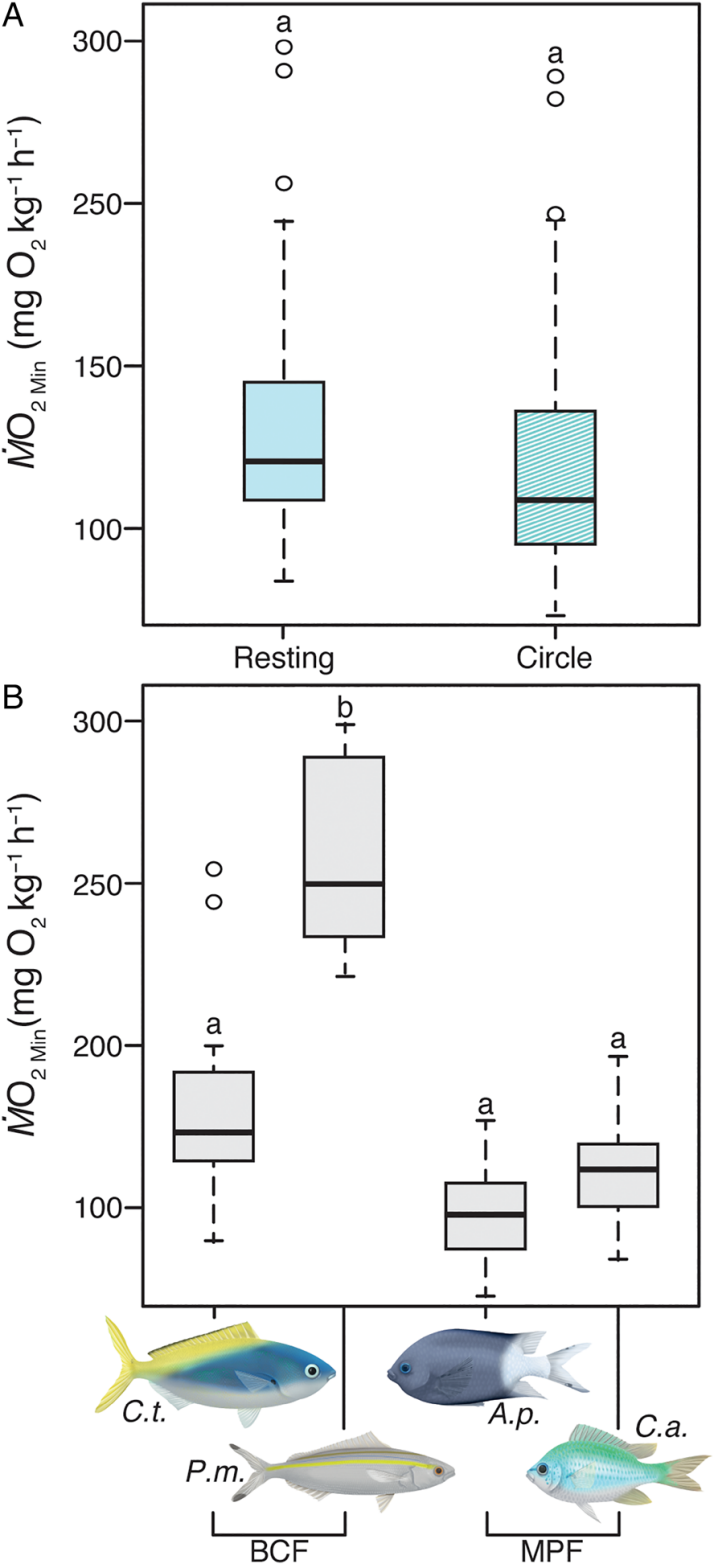
Boxplots showing median and inter-quartile range of (**A**) standard metabolic rates (SMR; estimated from lowest value of oxygen consumption rate, $\dot{M}O_{2\mathrm{Min}}$; see Materials and methods for further details) using two respirometry methods, a resting respirometer (Resting) and a circular chamber with a stir bar (Circle), for all species combined that completed at least one method and (**B**) SMR for all fishes that completed at least one method. *Caesio teres* (*C.t.*) and *Pterocaesio marri* (*P.m.*) are body–caudal fin (BCF) swimmers. *Acanthochromis polyacanthus* (*A.p.*) and *Chromis atripectoralis* (*C.a.*) are median–paired fin (MPF) swimmers. Same letters indicate no significant differences (α = 0.05).

Estimates of SMR did not differ between the two respirometry methods assessed, which included the resting and circular chamber protocols (LMM, *F*_1,32_ = 0.38, *P *= 0.54; Fig. 4A; Table 1). However, there were noticeable differences between species (LMM, *F*_3,33_ = 9.28, *P *< 0.001; Fig. 4B), with *P. marri* having a significantly higher SMR than the other three species. The effect of method was the same across species (species* *× method interaction not significant, *F*_3,29_ = 1.92, *P *= 0.15; online supplementary material, Fig. S3B; Table 1).

Body–caudal fin swimming fishes exhibited a significantly higher absolute *U*_crit_ (in centimetres per second) than MPF swimming fishes (LM, *F*_1,31_ = 16.1, *P *< 0.001; Table 2), and absolute *U*_crit_ differed slightly among species within swimming mode (LM, *F*_2,31_ = 1.1, *P *= 0.03; Table 2). Values of absolute and relative *U*_crit_ for all species are presented in Table 2.

**Table 2:** Absolute and relative critical swimming speed by species and swimming mode for all fishes that completed the swimming trials

Mode	Species	*U*_crit_ (absolute; cm s^−1^)		*U*_crit_ (relative; body lengths s^−1^)	
		Mean	SEM	Mean	SEM
BCF	*Pterocaesio marri*	82.05^a^	5.10	10.10^ab^	1.04
BCF	*Caesio teres*	83.96^a^	3.20	10.13^a^	0.30
MPF	*Acanthochromis polyacanthus*	63.12^b^	1.76	8.43^a^	0.19
MPF	*Chromis atripectoralis*	75.57^a^	3.41	12.10^b^	0.50

Swimming modes included body–caudal fin (BCF) and median–paired fin (MPF) swimming. *U*_crit_, critical swimming speed. Sample sizes are as in Table 1. Common letters indicate no significant differences (α = 0.05).

## Discussion

This study demonstrates the importance of choosing the appropriate method when estimating metabolic rates in fishes. Swimming respirometry involves incrementally increasing swimming speeds over several hours until the animal reaches a maximal swimming speed that is unsustainable. This technique has been traditionally referred to as the most accurate means of estimating MMR for steady/prolonged swimmers (Steffensen *et al*., 1984; Farrell and Steffensen, 1987; Plaut, 2001). The incremental increase in speed allows for the routine gait of a species to be used at low speeds, with transitions to caudal-assisted (*U*_p-c_ for MPF swimmers; Johansen and Jones, 2011; Binning *et al*., 2014; Johansen *et al*., 2015) and/or anaerobic burst-and-coast swimming (*U*_burst_; Svendsen *et al*., 2010) as speeds increase, during which time substrate limitations will result in fibre and therefore muscle exhaustion. Additionally, MMR is estimated directly while the fish is maximally exercised. Indeed, we found that swimming respirometry consistently gave the highest estimates of MMR in both BCF and MPF swimmers. The two short-duration protocols, where fish are challenged for 3 min with 1 min air exposure (chase protocol) or 5–7 min (circular chamber protocol) both underestimated MMR by as much as 38% for a single species compared with swimming respirometry (online supplementary material, Table S1). This may be because MPF swimmers would be forced to transition immediately to their final swimming mode (caudal burst swimming), which involves different kinematics from their routine, labriform swimming mode (Webb, 1975, 1984; Blake, 2004), in order to undergo the challenge. In contrast, for BCF swimmers, the kinematics of movement between routine and burst swimming are very similar. Therefore, in theory, all methods for estimating MMR should have resulted in similar values for BCF swimmers and different values for MPF swimmers, but this prediction did not hold.

For these prolonged swimmers, swimming mode did not influence the best choice of respirometry method used to estimate MMR. However, other aspects may be important when considering respirometry methods. For example, the duration of the challenge and protocol may play a key role (e.g. incremental increases in speed over several hours in the swimming protocol vs. a few minutes in the chase and circular chamber protocols). Even within a long-duration protocol, such as with swimming respirometry, the time between the incremental increases in water velocity can affect how fish respond in terms of oxygen consumption rates (Kiceniuk and Jones, 1977; Farrell, 2007). The size of the animal and flow encountered in their natural habitat should also be taken into consideration (Nelson, *et al.*, 2002). It should be noted that the effect on unsteady swimming species that perform poorly in a swim tunnel (e.g. burst swimmers and ambush predators; Killen *et al*., 2007) has not been thoroughly tested across species (see examples for cod in Soofiani and Priede, 1985; Reidy *et al*., 1995).

### Locomotory modes and duration of physiological performance in fishes

Swimming is characterized by the structures required (e.g. fins and muscles) and duration. Body–caudal fin swimmers rely on caudal body musculature and a caudal fin to generate thrust, which can support steady movements over prolonged periods of time in some species (Blake, 1983) and unsteady movements over short periods of time in others (e.g. ambush predators; Fig. 2). In contrast, MPF swimmers employ a lift-based (wing) or rowing (oar) paired fin movement, while the body remains rigid to reduce drag and save energy (Fig. 2; Webb, 1984; Blake, 2004). Median–paired fin swimmers are generally optimized for maintaining position, hovering and manoeuvring in complex environments, especially for those with more rounded fins, and were not traditionally viewed as high-performance endurance swimmers (but, see Fulton *et al*., 2013; Binning and Roche, 2015). Sustained swimming (via BCF or MPF) is generally considered to last >200 min; in contrast, prolonged swimming (20 s to 200 min) and burst-and-coast swimming (<20 s) both end in fatigue (reviewed by Blake, 2004; but also see Sfakiotakis *et al.*, 1999; Korsmeyer *et al*., 2002). Sustained or endurance swimming, which can be measured using traditional swimming protocols, is predominantly aerobic, using red muscle and not limited by fuel supply because lipids, proteins and carbohydrates can all be oxidized aerobically (Peake and Farrell, 2004; Fangue *et al*., 2008). Then, as fish approach maximal swimming speeds, they recruit fast-twitch, glycolytic, white muscle, which generates more thrust (Rome *et al*., 1996; Korsmeyer *et al*., 2002; Peake and Farrell, 2004; Svendsen *et al*., 2010). Consequently, the short-duration protocols (chase or circular chamber) may be exploiting only this final burst-and-coast gait, explaining why MMR can be significantly underestimated.

As species can be optimized for steady or short-term burst performance, the most relevant assessments of metabolic performance during swimming must consider the swimming behaviour of the species in their natural habitat and the duration over which it occurs. For instance, some species cannot or will not swim for extended periods and may therefore not be amenable to a protocol where water velocity is incrementally increased over several hours to assess swimming performance and estimate MMR (Reidy *et al*., 1995; Claireaux *et al*., 2006; Jordan and Steffensen, 2007; Killen *et al.*, 2007). We investigated steady/prolonged swimmers from both BCF and MPF swimming modes and found that a longer duration protocol provides the most accurate estimates of MMR for these species that can sustain high swimming speeds for long periods.

Other methods for estimating MMR may provide more cost- and time-effective alternatives to swimming respirometry. In chase protocols, the fish is forced to use burst, anaerobic swimming until fatigue. However, it is important to consider that after an exhaustive chase challenge, $\dot{M}O_{2}$ measurements come from excess post-exercise oxygen consumption (EPOC) or repayment of oxygen debt incurred from anaerobic metabolism. There are at least two assumptions underpinning this protocol. The first assumption is that EPOC represents the highest rate of oxygen consumption and is therefore equal to $\dot{M}O_{2\mathrm{Max}}$ and thus estimates of MMR. The second assumption is that EPOC occurs immediately after the exercise challenge, and recovery takes long enough for a reliable estimate to be obtained. Neither of these assumptions has been rigorously verified across species (see example for barramundi, *Lates calcarifer*, by Norin and Clark, 2016). If EPOC occurs immediately after a challenge, but recovery is quick, there is a risk of missing the measurements between the time the fish is challenged and when the measurement begins, as with the chase protocol. Theoretically, chase protocols may also delay EPOC by elevating plasma glucose concentrations and delaying glycogen resynthesis and lactate clearance because of stress (Milligan *et al*., 2000; Peake and Farrell, 2004). In both of these situations, MMR may be underestimated. Nonetheless, exhaustive chase methods may be ideal for unsteady swimmers, such as ambush predators, and future studies should compare methods in such species and others with more burst-type lifestyles.

Recent studies have used another type of exhaustive chase method with a circular chamber protocol (Nilsson *et al.*, 2009; Donelson *et al.*, 2010, 2011; Gardiner *et al.*, 2010; Couturier *et al*., 2013; Rummer *et al*., 2013; Pope *et al.*, 2014) as an alternative to swimming respirometry (e.g. Trappett *et al*., 2013). However, we generally do not recommend the use of a circular chamber, especially if the goals of the study are to compare estimates of AS or MMR with other studies where other methods have been used or to report some measure of swimming speed. This recommendation is based on several lines of reasoning, as follows: (i) velocity across the diameter of the circular respirometer increases significantly towards the edges of the vortex (also mentioned by Nilsson *et al.*, 2007), meaning that the swimming speed of the individual cannot be measured accurately or compared reliably within/among studies or to field conditions; (ii) fish are constantly swimming in either a clockwise or anticlockwise direction, which will result in an imbalanced use of their musculature, may prevent recruitment of all red muscle and therefore achievement of maximal aerobic metabolism, and is likely to cause premature fatigue of right- or left-side muscles, but not complete exhaustion; and (iii) because measurements are made continuously during the exercise period, the flush pump cycle, which periodically provides the fish with clean, well-aerated seawater, cannot be started until after the fish has fatigued, risking chamber O_2_ concentrations falling below 80% and initiating a hypoxic stress response (Hughes, 1973; Tetens and Lykkeboe, 1985; Boutilier *et al*., 1988).

Although the circular chamber protocol cannot be compared directly with swimming respirometry and does not allow manual chasing for species that will not swim for prolonged periods (e.g. serranids and gadids), there may be situations where this protocol can be used effectively. Advantages include the following: (i) fish are not moved between chambers, and the investigator is therefore less likely to ‘miss’ the $\dot{M}O_{2\mathrm{Max}}$ values because O_2_ is being monitored continuously; and (ii) unlike chase methods, the water velocity can be increased incrementally to allow fish to transition between gaits gradually in order to achieve maximal swimming speeds. As a result, this protocol may work well for some species that cannot be manipulated easily by the researcher or are too delicate for manual chasing protocols (e.g. larval fishes, as in Nilsson *et al*., 2007). However, researchers should be aware of the difficulties in comparing and interpreting results obtained with a circular chamber protocol and use this technique only if other options have proved unsuccessful or are not feasible.

The ecology of a species may also be important when assessing SMR. Some species that are optimized for fast, sustained swimming (e.g. mackerel and tuna) have difficulty maintaining very low speeds and can exhibit highly variable oxygen consumption rates owing to stress while trying to maintain position (Korsmeyer and Dewar, 2001). Additionally, species using ram ventilation (e.g. tuna and sharks) have to swim continuously to survive; therefore, it may be nearly impossible to estimate SMR accurately using conventional resting methodologies (Clark and Seymour, 2006). Rather, SMR in these species should be estimated indirectly by extrapolation of the non-linear $\dot{M}O_{2}$–swimming speed relationship using swimming respirometry (Bushnell *et al*., 1994; Reidy *et al*., 2000; Korsmeyer and Dewar, 2001; Korsmeyer *et al*., 2002; Clark and Seymour, 2006; Roche *et al*., 2013; Binning *et al*., 2014). In other fishes, however, SMR can be estimated directly in a non-swimming, relaxed state using a resting respirometer (reviewed by Clark *et al*., 2013 and Svendsen *et al*., 2016; but also see Steffensen, 1989; Roche *et al*., 2013). Indeed, the two resting protocols trialled (post-chase resting chamber and circular chamber) provided consistent estimates of SMR in the present study.

### Conclusions and recommendations

In recent decades, there has been a focus on studies linking swimming mode, physiological performance and environmental stress, emphasizing the importance of choosing appropriate methods for addressing a specific research question. The present study highlights that swimming respirometry appears to provide the most accurate estimates of MMR in fish that are steady/prolonged swimmers, regardless of swimming mode. In addition, swimming respirometry provides additional valuable information about swimming performance beyond critical swimming speed and MMR, such as gait transitions, burst speed, optimal swimming speed and cost of transport, all of which are ecologically relevant and could be influenced by changing environmental conditions. If swimming respirometry is not possible for fishes that are good steady/prolonged swimmers, short-duration chase or circular chamber protocols could be used, but with caution, because they may significantly underestimate MMR and therefore AS (see also Roche *et al.*, 2013). Fish that predominantly use burst swimming (e.g. ambush predators) or unsteady MPF swimming (e.g. labriforms that use ‘rowing’ instead of ‘flapping’; see Binning and Roche, 2015) may do better in short-duration challenges, but these predictions remain to be investigated. Nevertheless, the two short-duration alternative methods investigated here did provide direct measurements of $\dot{M}O_{2\mathrm{Min}}$ and therefore provided equally reliable SMR estimates.

From a technical perspective, both swimming respirometry and the chase protocol can easily be standardized and automated (e.g. using commercially available software) to maintain oxygen concentrations above 80% saturation at all times while calculating oxygen consumption rates at regular intervals (Svendsen *et al.*, 2016). These tools can ease user implementation and could minimize the subjectivity or bias in data collection (i.e. manually selected oxygen consumption rate slopes, duration of slope to use, *r*^2^ of slopes, etc.). The circular chamber, however, cannot be automated as easily because the point at which maximal swimming performance and fatigue will occur cannot be anticipated easily. Some methods may also be financially less costly (e.g. chase protocol), and with shorter protocols, more fish can be tested in a limited amount of time. However, if the method chosen is not a good match for the species, then the quality of measurements will be sacrificed for quantity. In particular, this may pose a significant problem if the goal of the study is to estimate AS, because our data suggest that using chase or circular chamber protocols for prolonged swimmers can underestimate MMR (online supplementary material, Table S1) and therefore AS. Additionally, because general correction factors cannot be applied to results *post hoc* owing to inter-individual, species-specific and/or temperature-driven differences in the degree of underestimation, caution is warranted if MMR and aerobic scope estimates are compared across studies that use different methods. Indeed, this has been a topic that has recently received a lot of attention and discussion, given that these measurements are being extensively used to understand organismal responses to contemporary issues, such as climate change (see Special Issue: Metabolic Rate in Fishes. Definitions, Methods and Significance for Conservation Physiology, volume 88, in *Journal of Fish Biology*). Ultimately, from our findings, we suggest that researchers consider the following factors: (i) the swimming mode/duration/lifestyle of the species; (ii) the constraints of the methods available; and (iii) potentially cross-validating results between methods to determine the most appropriate method for the species of interest.

## Supplementary material

Supplementary material is available at *Conservation Physiology* online. The data for this study are also publicly archived on the repository figshare. doi: 0.6084/m9.figshare.2060022: https://figshare.com/articles/Methods_matter_Considering_locomotory_mode_and_respirometry_technique_when_estimating_metabolic_rates_of_fishes/2060022?

## Funding

Funding for this project was provided by an Australian Research Council Super Science Fellowship and Australian Research Council early career Discovery Fellowship (J.L.R.), the Australian Research Council Centre of Excellence for Coral Reef Studies (J.L.R., D.G.R., S.A.B.) the Australian National University (S.A.B., D.G.R.), the Natural Sciences and Engineering Research Council of Canada (S.A.B., D.G.R.), Total Diving in Montréal (D.G.R., S.A.B.) and the Ian Potter Doctoral Fellowship at Lizard Island (a facility of the Australian Museum; D.G.R., S.A.B.).

## Supplementary Material

Supplementary Data
