# Supplementary material for: Methods matter: considering locomotory mode and respirometry technique when estimating metabolic rates of fishes
Source: Conserv Physiol. 2016 Mar 23;4(1):cow008. doi: 10.1093/conphys/cow008 (PMC4922262; doi:10.1093/conphys/cow008)
Supplement: Supplementary Data [file supp_4_1_cow008__index.html]

Supplementary Data 

# Methods matter: considering locomotory mode and respirometry technique when estimating metabolic rates of fishes

## Supplementary Data

Supplementary Data

- Supplementary Data - Docx file
- Supplementary Figure 1 - tif file
- Supplementary Figure 2 - tif file
- Supplementary Figure 3 - tif file
