# Supplementary material for: Methods matter: considering locomotory mode and respirometry technique when estimating metabolic rates of fishes
Source: Conserv Physiol. 2016 Mar 23;4(1):cow008. doi: 10.1093/conphys/cow008 (PMC4922262; doi:10.1093/conphys/cow008)
Supplement: Supplementary Data [file supp_cow008_cow008supp.docx]

**Supplemental Material**

Methods matter: Considering locomotory mode and respirometry technique when estimating metabolic rates of fishes

Jodie L. Rummer^1,^*, Sandra A. Binning^2,3^, Dominique G. Roche^2,3^, and Jacob L. Johansen^1,4^

The data for this study are also publicly archived on the repository figshare.

doi: 10.6084/m9.figshare.2060022

Link: https://figshare.com/articles/Methods_matter_Considering_locomotory_mode_and_respirometry_technique_when_estimating_metabolic_rates_of_fishes/2060022?

**Table S1.** The % by which the exhaustive chase (Chase) and circular swim chamber (Circle) methods underestimate maximum metabolic rate (MMR) when compared to estimates derived from swimming respirometry (Swim).

|  |  | **MMR underestimate** | |
| --- | --- | --- | --- |
| **Species** |  | **(%, compared to Swim)** | |
|  |  | **Chase** | **Circle** |
| *Pterocaesio marri* | Mean | -22.7 | -38.3 |
|  | S.E.M. | ±10.5 | ±2.5 |
|  |  |  |  |
| *Caesio teres* | Mean | -15.5 | -15.4 |
|  | S.E.M. | ±9.5 | ±5.6 |
|  |  |  |  |
| *Acanthochromis* | Mean | -35.3 | -21.6 |
| *polyacanthus* | S.E.M. | ±3.6 | ±7.0 |
|  |  |  |  |
| *Chromis atripectoralis* | Mean | -6.4 | -24.1 |
|  | S.E.M. | ±10.1 | ±6.5 |
| **Grand Mean** | | **-20.0** | **-24.9** |
|  | **S.E.M.** | **±6.1** | **±4.9** |

**Fig S1.** Top down view of the circular chamber respirometer, as in Fig. 1C, to scale. The gray shaded area indicates the working area of the chamber, i.e. the areas where the fish can swim in straight lines.

**Fig S2.** Representative traces depicting *Ṁ*O_2_ over time for (up to 13h) following either the chase (left column panels) or circle (right column panels) challenge (see Materials and Methods for further details) for all four species of fishes investigated, *Pterocaesio marri*, *Caesio teres*, *Acanthochromis polyacanthus*, and *Chromis atripectoralis* (respectively).

**Fig S3.** Results from the mixed-effects model computed using the R package effects (Fox *et al.,* 2014) for A) maximum metabolic rate (MMR) and B) standard metabolic rate (SMR), where species and respirometry method are fixed factors and individual is a random factor. N.B. Since these are graphical effect displays that account for blocking by individuals, the values do not match the means shown in Table 1.
