# Supplementary figures and images for: Methods matter: considering locomotory mode and respirometry technique when estimating metabolic rates of fishes
Source: Conserv Physiol. 2016 Mar 23;4(1):cow008. doi: 10.1093/conphys/cow008 (PMC4922262; doi:10.1093/conphys/cow008)

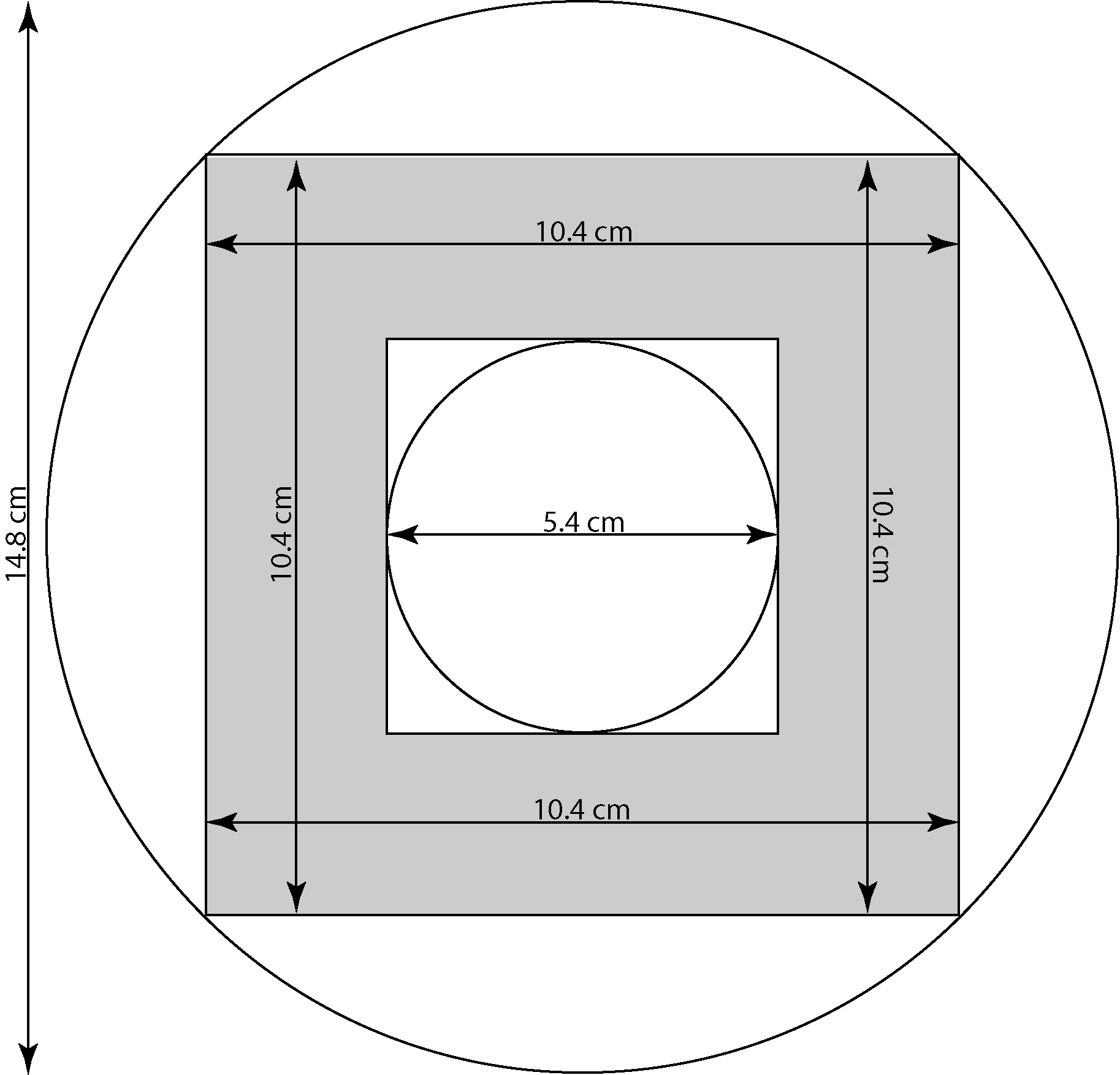

Supplement: Supplementary Data [file supp_cow008_cow008supp_fig1.tif]

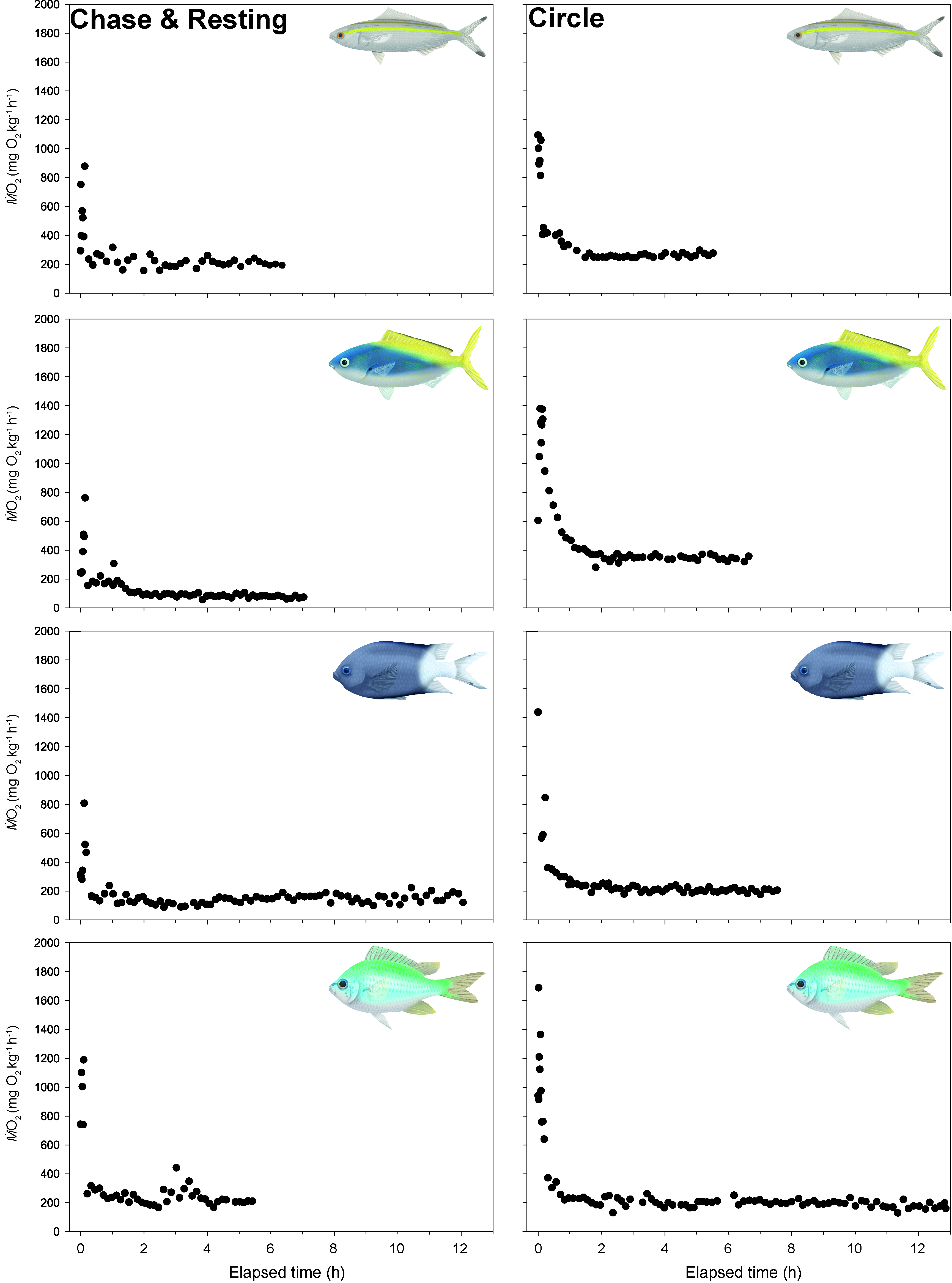

Supplement: Supplementary Data [file supp_cow008_cow008supp_fig2.tif]

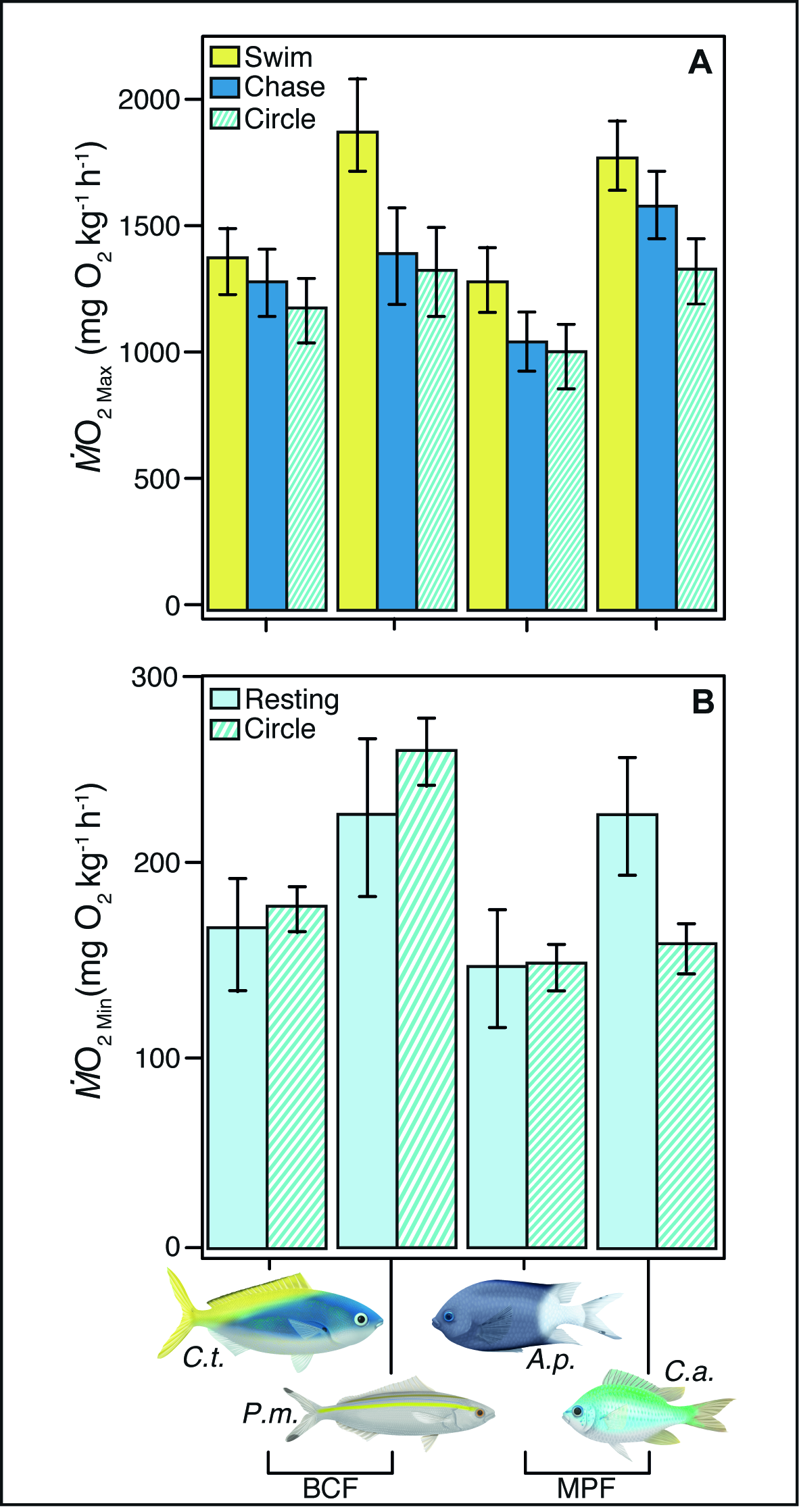

Supplement: Supplementary Data [file supp_cow008_cow008supp_fig3.tif]
